# Performance of SARS-CoV-2 antigen testing in symptomatic and asymptomatic adults: a single-center evaluation

**DOI:** 10.1186/s12879-021-06716-1

**Published:** 2021-10-18

**Authors:** Stephanie L. Mitchell, Steven Orris, Tanner Freeman, Megan C. Freeman, Michelle Adam, Meredith Axe, Jamie Gribschaw, Joe Suyama, Alejandro Hoberman, Alan Wells

**Affiliations:** 1 Department of Pathology, University of Pittsburgh, 3477 Euler Way, Clinical Laboratory Building-6th Floor, Pittsburgh, PA 15213 USA; 2 Division of General Internal Medicine, Center for Research On Health Care, Department of Medicine, University of Pittsburgh, Pittsburgh, PA USA; 3 Department of Pediatrics, Division of Infectious Diseases, University of Pittsburgh, Pittsburgh, PA 15213 USA; 4 Clinical Microbiology Laboratory, UPMC Hospital System, Pittsburgh, PA 15261 USA; 5 Department of Emergency Medicine, University of Pittsburgh, Pittsburgh, PA 15213 USA; 6 Department of Pediatrics, University of Pittsburgh, Pittsburgh, PA 15213 USA

**Keywords:** SARS-CoV-2, COVID-19, Antigen test, PCR, Asymptomatic, Symptomatic

## Abstract

**Background:**

Antigen testing offers rapid and inexpensive testing for SARS-CoV-2 but concerns regarding performance, especially sensitivity, remain. Limited data exists for use of antigen testing in asymptomatic patients; thus, performance and reliability of antigen testing remains unclear.

**Methods:**

148 symptomatic and 144 asymptomatic adults were included. A nasal swab was collected for testing by Quidel Sofia SARS IFA (Sofia) as point of care. A nasopharyngeal swab was also collected and transported to the laboratory for testing by Cepheid Xpert Xpress SARS-CoV-2/Flu/RSV RT-PCR (Cepheid).

**Results:**

Overall, Sofia had good agreement with Cepheid (> 95%) in adults, however was less sensitive. Sofia had a sensitivity of 87.8% and 33.3% for symptomatic and asymptomatic patients, respectively. Among symptomatic patients, testing > 5 days post symptom onset resulted in lower sensitivity (82%) when compared with testing within 5 days of symptom onset (90%). Of the four Sofia false-negative results in the asymptomatic cohort, 50% went on to develop COVID-19 disease within 5 days of testing. Specificity in both symptomatic and asymptomatic cohorts was 100%.

**Conclusions:**

Sofia has acceptable performance in symptomatic adults when tested < 5 days of symptom onset. Caution should be taken when testing patients with ≥ 5 days of symptoms. The combination of low prevalence and reduced sensitivity results in relatively poor performance of in asymptomatic patients. NAAT-based diagnostic assays should be considered in when antigen testing is unreliable, particularly in symptomatic patients with > 5 days of symptom onset and asymptomatic patients.

## Background

Throughout the SARS-CoV-2 pandemic, there has been a consistent need for rapid and accurate testing. Nucleic acid amplification test (NAAT)-based diagnostics, such as RT-PCR, remain the gold standard. In efforts to increase test capacity, antigen testing has been introduced as an alternative testing method. Currently, numerous antigen devices have been authorized for use by the FDA under Emergency Use Authorization (EUA) for testing in symptomatic patients. Advantages of antigen testing are certainly desirable- fast, less costly and near patient- and have been implemented in a variety of institutions, from universities to nursing homes. However, it is not without limitations, with the most significant limitation being reduced sensitivity as compared to NAAT-based assays. False-negative results can have compounding negative effects in terms of controlling and reducing community spread of SARS-CoV-2.

Infection with SARS-CoV-2 results in a variety of clinical presentations from no symptoms to fulminate pneumonia, where asymptomatic patients with SARS-CoV-2 infection often go undiagnosed or unrecognized, though the role these patients play in the spread of disease is still in question [1, 2]. Pre-symptomatic patients present their own unique set of challenges. Diagnosis typically occurs after symptom onset where the opportunity of viral transmission and spread may have already occurred [3–5]. Therefore, accurate detection of SARS-CoV-2 in pre-symptomatic individuals is of vital importance to controlling spread of COVID-19. Even though antigen testing is being widely used in a variety of settings, its performance remains understudied in a- and pre-symptomatic patients. Studies have shown reduced sensitivity of antigen testing in both symptomatic and asymptomatic populations but particularly among asymptomatic patients, with some reports as low as 40% even among those with known household contacts [6–8]. Sensitivity is further reduced when testing asymptomatic patients without known contacts [8], raising the question if antigen testing is a reliable method for use in this population.

Herein, we report the performance of the Quidel Sofia SARS Antigen FIA in a point of care setting for symptomatic and asymptomatic adult patients given the lack of performance data for this specific antigen test.

## Methods

### Ethics

All RT-PCR testing was performed as a part of routine clinical care, according to CLIA’88 regulations. This project was approved by the UPMC Quality Assurance Board and undertaken as a Quality Improvement Initiative and as such was not formally reviewed by the University of Pittsburgh Institutional Review Board. Informed verbal or written patient consent was waived as per the UPMC Quality Assurance Board. Patient data collected for this project were de-identified, obtained and stored securely in a REDCap database (NIH/NCATS UL1 TR000445) via HIPAA-compliant procedures. No administrative permission was required to access raw data for the study as approved by the UPMC Quality Assurance Board.

### Patient selection and sample collection

148 symptomatic and 144 asymptomatic adult patients, ≥ 18 years of age, presenting to outpatient care at either a UPMC outpatient COVID-19 testing facility or the University of Pittsburgh Health Services center in Pittsburgh, PA were included in the study. Patients that were < 18 years of age were excluded. Patients were classified as symptomatic or asymptomatic as per the physician order or chart review. Patients were considered symptomatic if they presented with any of the following symptoms: fever, cough, congestion, shortness of breath, headache, loss of taste/smell, sore throat, abdominal pain, vomiting, nausea, diarrhea, myalgia, fatigue and chills. Patients were classified as asymptomatic if they were without symptoms at time of testing. Testing was performed due to known exposure or because it was required for work, travel/re-entry, pre-procedural or other asymptomatic screening.

Two samples were collected from each patient included in the study. The first specimen, as per standard of care (SOC), was a nasopharyngeal (NP) swab that was collected and placed in viral transport media (VTM) (Remel M4-RT, Remel, San Diego, CA). NP samples were transported to the clinical microbiology laboratory for testing by SOC RT-PCR. A second specimen, nasal swab, was collected either by the healthcare provider (COVID testing site) or self-collected under the supervision of a healthcare provider (student health) for antigen testing. Direct antigen testing was performed at the site of collection (eg, testing center or student health). Testing was performed from January 2021 until March 2021.

### Antigen testing

The Quidel Sofia SARS IFA antigen assay (Sofia; San Diego, CA) is authorized by the FDA for qualitative detection of SARS-CoV-2 by detecting the presence of SARS-CoV-2 nucleocapsid antigen by a sandwich immunoassay methodology. Sofia was chosen for the study due to lack of performance data for this assay and easy access to Sofia instrument and tests. Sofia was performed directly on nasal swabs as per the manufacturer’s instructions [9]. Testing was performed as a point of care test at the site of collection by trained personnel as per CLIA’ 88 for waived laboratory testing. Collection site was a sample collection center for patients requiring testing for SARS-CoV-2.

### Nucleic acid testing

SOC PCR testing on NP swabs in VTM were performed on the Cepheid GeneXpert® Xpress SARS-CoV-2/Flu/RSV real-time RT-PCR assay (Cepheid; Sunnyvale, CA) [10], which is authorized by FDA for the qualitative detection of SARS-CoV-2. Sample was transported to the clinical laboratory and testing was performed on-site at the clinical laboratory as per the manufacturer’s instructions and according to CLIA’88 for moderate-high complexity testing. Testing was performed within 48 h of sample receipt. Quality control was performed as per the laboratory’s individual quality control plan and according to CLIA’88 requirements. Cepheid was the reference method for the study.

### Data collection and analysis

Patients were stratified into symptomatic or asymptomatic groups for data analysis. Review of the electronic medical record (EMR) was performed to confirm proper designation of specimens as symptomatic or asymptomatic and ensure exclusion of repeat specimens. Patient demographics, reason for testing and exposure history were collected. A positive exposure was defined as greater than 15 min of contact, without a mask, with an individual with a documented positive SARS-CoV-2 result. Twenty-four patients categorized as asymptomatic surveillance were excluded from clinical analysis as no medical note could be identified during the review of the electronic medical record. For symptomatic patients, the days from symptom onset until testing was also collected and grouped into < 5 days and ≥ 5 days. Performance of Sofia was determined by comparing to the results from Cepheid. Percent agreement, sensitivity, specificity, positive and negative predictive values (PPV and NPV) as well as overall prevalence were calculated using contingency tables. Prevalence was calculated based on Cepheid positivity as the gold standard. Positive and negative predictive values, factoring in prevalence for each cohort, were calculated as follows:

$${\text{PPV}}:{{\left( {{\text{sensitivity }} \times {\text{ prevalence}}} \right)} \mathord{\left/ {\vphantom {{\left( {{\text{sensitivity }} \times {\text{ prevalence}}} \right)} {\left( {\left( {{\text{sensitivity }} \times {\text{ prevalence}}} \right) \, + \, \left( {\left( {{1 }{-}{\text{ specificity}}} \right) \, \times \, \left( {{1 }{-}{\text{ prevalence}}} \right)} \right)} \right)}}} \right. \kern-\nulldelimiterspace} {\left( {\left( {{\text{sensitivity }} \times {\text{ prevalence}}} \right) \, + \, \left( {\left( {{1 }{-}{\text{ specificity}}} \right) \, \times \, \left( {{1 }{-}{\text{ prevalence}}} \right)} \right)} \right)}}$$

$${\text{NPV}}:{{\left( {{\text{specificity }} \times \, \left( {{1 }{-} {\text{prevalence}}} \right)} \right)} \mathord{\left/ {\vphantom {{\left( {{\text{specificity }} \times \, \left( {{1 }{-} {\text{prevalence}}} \right)} \right)} {\left( {\left( {{\text{specificity }} \times \, \left( {{1 }{-} {\text{prevalence}}} \right)} \right) \, + \, \left( {\left( {{1 }{-}{\text{ sensitivity}}} \right) \, \times {\text{prevalence}}} \right)} \right)}}} \right. \kern-\nulldelimiterspace} {\left( {\left( {{\text{specificity }} \times \, \left( {{1 }{-} {\text{prevalence}}} \right)} \right) \, + \, \left( {\left( {{1 }{-}{\text{ sensitivity}}} \right) \, \times {\text{prevalence}}} \right)} \right)}}$$

For asymptomatic patients, discrepancies between Sofia and Cepheid were clinically adjudicated by in-depth chart review. Statistics including averages, range and Student’s T-Test was determined using Microsoft Excel. T-test was used for comparison of C_T_ values; *p* < 0.05 were considered statically significant.

### Statistical considerations

For assessing diagnostic tests, the general recommendation is to include 200–300 patients for appropriate power to observe statistical significance [11]. However, prevalence is one factor in calculating the minimum sample size required, along with assay sensitivity and specificity. Based on the observed prevalence, our study is sufficiently powered for the symptomatic cohort but may be underpowered for the asymptomatic cohort in determining antigen test performance (eg, sensitivity and specificity).

## Results

**Fig. 1:**
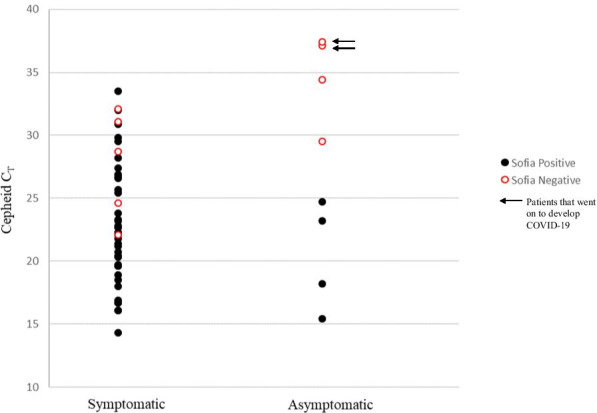
Correlation of Cepheid Positives with Sofia Results. Cepheid C_T_ values for positive samples were plotted against Sofia results (positive or negative). Black closed circles = Cepheid and Sofia positive; Red open circles = Cepheid positive and Sofia negative; arrows denote the two asymptomatic patients that went on to develop COVID-19 disease

During the study period, the prevalence of COVID-19 in our cohorts, as determined by Cepheid positivity, was 27.7% for symptomatic patients and 4.2% for asymptomatic patients. In general, the Sofia performed well with an overall percent agreement of 96.6% for symptomatic patients and 97.2% for asymptomatic patients (Table 1A and B). For symptomatic patients, overall sensitivity was 87.8%, with five false-negative (FN) results by Sofia (12.2% FN). Of the five FN, the cycle threshold (C_T_), which is often used as a loose semi-quantitation of sample viral load, ranged from 22.1 to 32.1 and spans what is generally considered to be strongly to moderately positive. For all Cepheid positives in the symptomatic cohort, the average C_T_ was 23.1 (range of 14.2–33.5; Fig. 1). Comparatively, the average C_T_ the Sofia FN was 27.7, which barely missed statistical significance (*p* = 0.05; data not shown). The NPV and PPV, when taking into account prevalence in our symptomatic population, was 95.5% and 100%, respectively. When Sofia performance was stratified by number of days from symptom onset to testing (< 5 or ≥ 5 days), sensitivity was better when testing at < 5 days of symptom onset (90% for < 5 days vs 81.8% for ≥ 5 days), thus resulting in a slight reduction in NPV for those tested at ≥ 5 days of symptom onset (Table 1A).

**Table 1:** Performance of Sofia stratified by days of symptoms

Sofia performance	No. days from symptom onset prior to test		All patients
	< 5 days	≥ 5 days	
(A) Symptomatic Patients. Symptomatic patients were stratified as < 5 days or ≥ 5 days of symptoms prior to receiving testing			
% Agreement	101/104 (97.1%)	42/44 (95.5%)	143/148 (96.6%)
Sensitivity	27/30 (90%)	9/11 (81.8%)	36/41 (87.8%)
Specificity	74/74 (100%)	33/33 (100%)	107/107 (100%)
PPV	100%	100%	100%
NPV	96.1%	94.3%	95.5%

Sofia performance	All asymptomatic patients		
(B) Asymptomatic Patients. PPV and NPV values were calculated with the respective prevalence for each group			
% Agreement	140/144 (97.2%)		
Sensitivity	2/6 (33.3%)		
Specificity	138/138 (100%)		
PPV	100.0%		
NPV	97.2%

For asymptomatic patients, Sofia’s overall sensitivity was 33.3%, with 4 FN results (66.7% FN; Table 1B). Of the 4 FN, the average C_T_ was 34.6 with a range of 29.5–37.4 (Fig. 1), with 3 of the 4 FN demonstrating C_T_ > 33, which is considered weakly positive. For all Cepheid positives in the asymptomatic cohort, the average C_T_ was 30.2 with a range of 15.4–37.4 and was statistically different from the average FN C_T_ value (*p* = 0.002; Fig. 1). The NPV and PPV in the asymptomatic cohort was determined to be 97.2% and 100%, respectively, when accounting for a prevalence of 4.2%. No false-positives were observed during our study for either population, resulting in a specificity of 100% (Table 1).

Average age of symptomatic patients tested was 44 years. Of the symptoms recorded, just above half of the patients reported cough and congestion, with headache, sore throat, myalgia and fatigue being the next most common symptoms (Table 2A). Interestingly, fever was only reported in 19% of patients. Overall, patients reported having a total of 3–4 symptoms. Twenty and nine tenths percent (20.9%) of patients had a known exposure and on average, testing was performed at day 5 post-exposure (Table 2A). However, the majority of patients were tested after 3–4 days of symptoms (Table 2A). Average age of asymptomatic patients tested was 38 years. The most common reason, if recorded, for asymptomatic testing was other asymptomatic surveillance, with pre-surgical procedure screening being second. Interestingly, testing of asymptomatic individuals with a known exposure only represented 5.6% of our population (Table 2B). However, those who had a known exposure received testing on average at day 2–3 post-exposure as opposed to day 5, as seen in our symptomatic cohort (Table 2).

**Table 2:** Patient Demographics and Test Characteristics

Symptomatic	Total	Cepheid +	Cepheid −	Sofia +	Sofia −
(A) Symptomatic					
n	148	41	107	36	112
Average age, yrs (range)	44.1 (18–83)	46.3 (18–83)	43.3 (20–76)	47.2 (20–83)	43.2 (18–76)
Sex, male	42 (28.4%)	21 (51.2%)	21 (19.6%)	18 (50%)	24 (21.4%)
Symptoms					
Fever	29 (19%)	15 (36.6%)	14 (13.1%)	11 (30.6%)	18 (16.1%)
Cough	76 (51.3%)	26 (63.4%)	50 (46.7%)	22 (61.1%)	54 (48.2%)
Congestion	77 (52.0%)	26 (63.4%)	51 (47.6%)	24 (66.7%)	53 (47.3%)
Shortness of breath	14 (9.5%)	4 (9.8%)	10 (9.3%)	4 (11.1%)	10 (8.9%)
Headache	45 (30.4%)	11 (26.8%)	34 (31.8%)	10 (27.8%)	35 (31.3%)
Sore throat	49 (33.1%)	9 (22.0%)	40 (37.4%)	8 (22.2%)	41 (36.6%)
Loss of taste	17 (11.5%)	10 (24.4%)	7 (6.5%)	9 (25.0%)	8 (7.1%)
Loss of smell	18 (12.2%)	10 (24.4%)	8 (7.5%)	9 (25.0%)	9 (8.0%)
Abdominal pain	5 (3.4%)	1 (2.4%)	4 (3.7%)	1 (2.8%)	4 (3.6%)
Nausea	12 (8.1%)	6 (14.6%)	6 (5.6%)	5 (13.9%)	7 (6.3%)
Vomiting	8 (5.4%)	2 (4.9%)	6 (5.6%)	2 (5.6%)	6 (5.4%)
Diarrhea	14 (9.5%)	3 (7.3%)	11 (10.3%)	3 (8.3%)	11 (9.8%)
Myalgia	32 (21.6%)	9 (22.0%)	23 (21.5%)	9 (25.0%)	23 (20.5%)
Fatigue	31 (20.9%)	6 (14.6%)	25 (23.3%)	4 (11.1%)	27 (24.1%)
Other (Chills)	24 (16.2%)	9 (22.0%)	15 (14.0%)	9 (25.0%)	15 (13.4%)
Total number of symptoms, average (range)	3.0 (1–7)	3.6 (1–7)	2.8 (1–7)	3.6 (1–7)	2.9 (1–7)
No. days of symptoms prior to test, average (range)	3.6 (1–7)	3.8 (1–7)	3.5 (1–7)	3.7 (2–7)	3.6 (1–7)
Known exposure	31 (20.9%)	12 (29.3%)	19 (17.7%)	11 (30.6%)	20 (17.9%)
No. days post-exposure until test, average (range)	5.3 (1–12)	4.8 ( 1–7)	5.8 (1–12)	5.1 (1–7)	5.5 (1–12)

Asymptomatic	Total	Cepheid +	Cepheid −	Sofia +	Sofia −
(B) Asymptomatic.					
n	144	6	138	2	142
Average age, years (range)	38.6 (17–80)	46.7 (28–68)	38.2 (17–80)	50.5 (34–67)	38.8(17–80)
Sex, male*	51 (41.1%)	2 (33.3%)	49 (41.5%)	1 (50.0%)	50 (41.0%)
Reason for testing					
Exposure	8 (5.6%)	2 (33.3%)	6 (4.3%)	0 (0%)	8 (5.6%)
Required for work	3 (2.1%)	1 (16.7%)	2 (1.4%)	0 (0%)	3 (2.1%)
Required for travel/Re-entry	10 (6.9%)	0 (0%)	10 (7.2%)	0 (0%)	10 (7.0%)
Required for procedure	33 (22.9%)	1 (16.7%)	32 (23.2%)	1 (50%)	32 (22.5%)
Asymptomatic Surveillance	47 (32.6%)	0 (0%)	47 (34.1%)	0 (0%)	47 (33.1%)
Unknown	43 (29.9%)	2 (33.3%)	41 (29.7%)	1 (50%)	42 (29.6%)
Known exposure	7 (4.9%)	2 (33.3%)	5 (3.6%)	0 (0%)	7 (5.0%)
No. days post-exposure to test, average (range)	3.4 (1–7)	2.5 (1–4)	4.0 (1–7)	N/A	3.4 (1–7)

*Total analyzed is 122 due to no sex identification denoted in the chart

Of the four Sofia FN observed in the asymptomatic population, additional chart review was performed for clinical resolution. One patient did not have an encounter note near the test date in our EMR system and was therefore excluded from clinical assessment. One patient required testing for work, was asymptomatic and had no known prior COVID-19 disease. The C_T_ value for this patient’s sample was > 35, where antigen testing has high likelihood to be falsely-negative [12]. The patient had a repeat COVID-19 test at an outside facility approximately 1 month later for unknown reasons, which was negative. From this review, it remains unclear whether this weak positive represents sporadic detection of unrecognized previous COVID-19 disease, asymptomatic carriage or an analytical false-positive. Interestingly, two of the patients that were FN by Sofia (50%) had known exposures, no prior known COVID-19 disease and went on to develop symptoms and COVID-19 disease around 5 days after initial testing. C_T_ values for these two samples were > 35. These two cases likely represent detection of early SARS-CoV-2 infection and thus true positives by Cepheid.

## Discussion

Overall, performance of Sofia in both patient populations was good, with > 95% agreement with Cepheid. Despite reports in the literature and media of high false-positive results when using antigen testing, we did not observe any false-positives during our study. However, this may be due to the limited number of asymptomatic patients in our study, resulting in the inability of our data to accurately reflect the specificity of the antigen test. Sofia sensitivity was less than Cepheid, which was not unexpected, missing nine true positives; five in the symptomatic cohort and four in the asymptomatic cohort. Additionally, for symptomatic patients, sensitivity of Sofia further decreased when testing patients with ≥ 5 days from symptom onset. Given that the median time to symptom onset is approximately 5 days and is near the average time that patients receive testing, the lack of sensitivity of Sofia at this time point may result in higher false-negative rates for this subset of patients, especially if testing is delayed by one or two days [13]. Sofia’s reduced sensitivity at > 5 days is not surprising given this is a known limitation of the assay, which is stated in the Sofia package insert. However, our data further strengthens and highlights this important consideration, as this reduction in the performance of antigen tests is often overlooked in clinical practice and rarely mentioned in testing guidelines or recommendations. Efforts should be taken to better educate those using antigen testing of this limitation, with careful consideration when using Sofia, such that NAAT testing be performed initially or to confirm antigen negative results on patients who present ≥ 5 days from symptom onset.

Concerns have been raised regarding the use of antigen testing in asymptomatic patients. Our study provides additional insight into antigen test performance in this population, where the pre-test probability is low. Sofia has poor sensitivity (33.3%) when testing this population, which is similar to what is described in the literature to date. While our NPV was high, it is important to consider that NPV is highly dependent on the prevalence in the intended population and thus may differ for other institutions. Nevertheless, lower prevalence is expected in an asymptomatic population, as compared to symptomatic patients, and thus our data likely represents real-world test performance. Interestingly, 2 of the 4, were patients who had a known exposure and went on to develop COVID-19 disease within 5 days from testing. This suggests that these patients were indeed true positives that were detected by Cepheid but missed by Sofia. The Cepheid C_T_ values for these two samples were both > 35, where antigen testing is inadequate. Taking the C_T_ and clinical context together, this suggests that these patients were early in their infection and represent the pre-symptomatic patient population, which remain a crux in controlling further transmission and spread of SARS-CoV-2. Reliable results are paramount to aid in the rapid implementation of public health measures to help reduce spread of the virus in the community. Thus, for accurate detection of SARS-CoV-2 in potentially pre-symptomatic patients, with known exposures, our data suggests that testing should be NAAT-based. Additionally, these two examples should also give pause to whether the strength of C_T_ values can be associated with infectious or non-infectious states. C_T_ values alone cannot predict contagiousness and the results must be taken in context with the clinical picture, such as exposure date and history, co-morbidities and symptomology.

Taken together, antigen testing can help expand rapid and affordable SARS-CoV-2 testing. Most importantly, careful consideration should be taken as to when and on whom the test is used to ensure that results are reliable. For certain patient populations, such as asymptomatic patients or those who have been symptomatic for ^3^5 days, NAAT-based diagnostics have a clear advantage and should be strongly recommended for primary use or incorporated into well-designed reflex algorithms to ensure antigen results can be trusted and are actionable.

## Conclusions

Sofia has acceptable performance in symptomatic adults when testing is performed < 5 days of symptoms. Low prevalence and reduced sensitivity results in relatively poor performance in asymptomatic patients. NAAT-based diagnostic assays and practical reflex algorithms should be considered in when antigen testing is unreliable, particularly in symptomatic patients with > 5 days of symptom onset and asymptomatic patients.

## Data Availability

The datasets used and/or analyzed during the current study are available from the corresponding author on reasonable request. Request can be made to Dr. Stephanie L. Mitchell; slm198@pitt.edu.

## Abbreviations

IFA: Immunofluorescence assay
RT-PCR: Reverse transcriptase polymerase chain reaction
NAAT: Nucleic acid amplification test
EUA: Emergency Use Authorization
SOC: Standard of care
NP: Nasopharyngeal
VTM: Viral transport media
PPV: Positive predictive value
NPV: Negative predictive values
EMR: Electronic medical record
FN: False-negative
C_T_: Cycle threshold
